# Effect of antioxidants on dentinal tubular penetration of root canal sealers in sodium hypochlorite treated root canal dentin: a systematic review

**DOI:** 10.3389/fdmed.2026.1766826

**Published:** 2026-03-26

**Authors:** Radhika Vasudev, Tony Mathew, Nireeksha

**Affiliations:** Nitte (Deemed to be University), AB Shetty Memorial Institute of Dental Sciences (ABSMIDS), Department of Conservative Dentistry and Endodontics, Mangalore, India

**Keywords:** antioxidants, CLSM, dentinal tubular penetration, glutathione, sealer, sealer penetration, SEM, sodium hypochlorite

## Abstract

**Background:**

The long-term success of root canal treatment depends on the formation of an effective three-dimensional seal, which is strongly influenced by the ability of endodontic sealers to penetrate dentinal tubules and adapt closely to the root canal walls. Sodium hypochlorite (NaOCl), the most commonly used endodontic irrigant, produces oxidative alterations in dentin, including collagen degradation, reduced surface wettability, and residual free radical formation, which collectively compromise sealer penetration and interfacial integrity. The use of antioxidant agents has been used as a strategy to reverse these adverse effects and restore dentin surface properties.

**Objective:**

This systematic review aimed to assess the influence of various antioxidant agents on dentinal tubular penetration and interfacial adaptation of root canal sealers following NaOCl irrigation.

**Methods:**

A comprehensive electronic search of PubMed, Scopus, Web of Science, and Google Scholar was performed up to November 2025. *In vitro* studies evaluating NaOCl-treated dentin followed by antioxidant application and reporting sealer penetration or adaptation outcomes using confocal laser scanning microscopy (CLSM) or scanning electron microscopy (SEM) were included. Methodological quality and risk of bias were evaluated using a modified QUIN assessment tool.

**Results:**

From 216 initially identified records, four studies fulfilled the eligibility criteria. The antioxidants investigated included ascorbic acid, tannic acid, gallic acid, ellagic acid, sodium thiosulfate, and glutathione. All antioxidant-treated groups demonstrated greater dentinal tubular penetration and increased interfacial adaptation compared with NaOCl-only controls. Polyphenolic antioxidants, particularly gallic acid and ellagic acid, produced the greatest enhancement in penetration depth. Sodium thiosulfate significantly reduced interfacial gaps and voids, while glutathione improved sealer penetration in both resin-based and bioceramic sealers and was associated with increased bond strength.

**Conclusion:**

The available *in vitro* evidence indicates that antioxidant agents effectively mitigate NaOCl-induced oxidative changes in dentin, leading to improved sealer penetration and adaptation. Incorporating antioxidants as a final irrigant step may enhance obturation quality and potentially improve sealer peneteration; however, well-designed clinical studies are required to confirm these findings.

**Systematic Review Registration:**

https://doi.org/10.17605/OSF.IO/PBRQA.

## 1 Introduction

One of the most important aims of root canal treatment is to create a long-lasting, three-dimensional seal, which is mostly dependent on how endodontic sealers interact with the dentinal substrate. The long-term success of root canal obturation is improved by dentinal tubular penetration because it facilitates mechanical interlocking, improved sealer adaption, and the entombment of residual bacteria (1, 2). However, the physicochemical state of dentin after irrigation has a significant impact on sealer penetration efficacy.

Because of its strong antibacterial action and special ability to disintegrate organic tissue, sodium hypochlorite (NaOCl) is still the most popular endodontic irrigant (3). However, research over the past ten years indicates that NaOCl negatively alters the structure of root dentin. It denatures the collagen matrix, decreases microhardness, increases brittleness, and leaves behind an oxidized, oxygen-rich layer that prevents resin-based sealers from penetrating and polymerizing (4–6). NaOCl-treated dentin frequently exhibits destroyed tubules, collapsed collagen fibrils, and uneven surfaces that impede sealer penetration, according to studies that were included in this review (7–10).

These structural modifications adversely influence the action of resin-based sealers. Residual free radicals created by NaOCl competitively reduce the free-radical polymerization, which results in incomplete cure, reduced interfacial bonding, and increased microleakage (11). When dentin surface energy and moisture balance are changed by exposure to NaOCl, even bioceramic sealers—which rely less on polymerization—show reduced penetration (10).

The use of antioxidant compounds as a last step for irrigation has drawn more attention as a solution to these problems. Reactive oxygen species (ROS) produced by NaOCl gets neutralized by antioxidants, repairs dentin's redox state, rebuild collagen integrity, and enhance the surface characteristics required for sealer flow, wettability, and interfacial adaptability (1). Over the past decade, research has been done on a variety of antioxidants, such as biological antioxidants such as glutathione, inorganic antioxidants such as sodium thiosulfate, and polyphenolic antioxidants such gallic acid, tannic acid, and ellagic acid.

Strong radical-scavenging capabilities are usually exhibited by polyphenolic antioxidants. When compared to tannic acid and ascorbic acid, Christopher et al. found that 10% gallic acid has greatly increased RealSeal SE's dentinal tubular penetration (7). Due to its potent deoxidizing and collagen-stabilizing qualities, Veenakumari et al. discovered that ellagic acid significantly enhanced AH Plus penetration in the coronal third (8). Current studies demonstrating that polyphenols increase bonding and strengthen the dentin collagen support these conclusions (12, 13).

Additionally, inorganic antioxidants have had encouraging outcomes. After NaOCl irrigation, De Lima Dias-Junior et al. showed that 0.5% sodium thiosulfate greatly decreases the interfacial gaps and improves the AH Plus penetration (9). Recent research on adhesive dentistry has confirmed that sodium thiosulfate improves resin polymerization by quickly neutralizing oxidized chlorine species and removing NaOCl residues (14, 15).

Recent years have seen an increase in interest in biological antioxidants like glutathione. Glutathione significantly increased the push-out bond strength and dentinal penetration of epoxy resin and bioceramic sealers, as shown by Charulatha et al. (10). By eliminating ROS and reestablishing the chemical environment necessary for the best sealer performance, its method entails thiol–disulfide exchange.

The systematic review by Gascón et al. (1) emphasizes variations in antioxidant methods, such as concentration, application time, and compatibility with adhesive systems, even when individual studies demonstrate good results. In order to optimize clinical practices, their review highlights the necessity of organized evaluation.

In order to better understand the effects of different antioxidant agents on dentinal tubular penetration and sealer–dentin interface quality after NaOCl irrigation, the current systematic review focuses on four important *in vitro* antioxidant investigations.

## 2 Materials and methods

This systematic review was conducted in accordance with the Preferred Reporting Items for Systematic Reviews and Meta-Analyses (PRISMA) guidelines and was registered in the OSF Registries. OSF Registration DOI: 10.17605/OSF.IO/PBRQA.

### 2.1 Search strategies

A single reviewer carried out the literature search and screened all studies relevant to the topic up to 10 November 2025. The literature search was initially performed by a single reviewer and subsequently verified and assisted by two additional reviewers to confirm the appropriateness of the search strategy and the completeness of the retrieved records as summarised in Table 1.

**Table 1:** Search strategy.

Database	Search strategy/query used	Results retrieved
PubMed (Search 1)	(“antioxidant” OR “sodium thiosulfate” OR “glutathione” OR “gallic acid” OR “N-acetyl cysteine” OR “maleic acid” OR “EDTA”) AND (“sodium hypochlorite” OR “NaOCl”) AND (“dentinal tubule penetration” OR “interfacial adaptation” OR “sealer penetration”) AND (“*in vitro*” OR “CLSM” OR “SEM”)	15 results
PubMed (Search 2)	(“antioxidant"[All fields] OR “sodium thiosulfate"[ All fields] OR “glutathione"[ All fields] OR “gallic acid"[ All fields] OR “N-acetyl cysteine"[ All fields] OR “maleic acid"[ All fields] OR “EDTA"[ All fields] OR “ethylenediaminetetraacetic acid"[ All fields]) AND [“sodium hypochlorite"[ All fields] OR “NaOCl"[ All fields]] AND (“dentinal tubule penetration"[ All fields] OR “dentinal tubular penetration"[ All fields] OR “interfacial adaptation"[ All fields] OR “sealer penetration"[ All fields]) AND (“*in vitro*"[ All fields] OR “confocal laser scanning microscopy"[ All fields] OR “CLSM"[ All fields] OR “scanning electron microscopy"[ All fields] OR “SEM"[ All fields])	25 results
Web of science	(“antioxidant” OR “sodium thiosulfate” OR “glutathione” OR “gallic acid” OR “N-acetyl cysteine” OR “maleic acid” OR “EDTA”) AND (“sodium hypochlorite” OR “NaOCl”) AND (“dentinal tubule penetration” OR “interfacial adaptation” OR “sealer penetration”) AND (“*in vitro*” OR “CLSM” OR “SEM”)	44 results
Scopus	(“antioxidant” OR “sodium thiosulfate” OR “glutathione” OR “gallic acid” OR “N-acetyl cysteine” OR “maleic acid” OR “EDTA”) AND (“sodium hypochlorite” OR “NaOCl”) AND (“dentinal tubule penetration” OR “interfacial adaptation” OR “sealer penetration”) AND (“*in vitro*” OR “CLSM” OR “SEM”)	29 results
Google scholar	Antioxidants, dentinal tubular penetration	103 results

Databases from the year 2015 to 2025 were searched and filter was applied to articles in English. The databases searched included PubMed (MEDLINE), Scopus, Web of Science, and Google Scholar, and each was examined thoroughly. The literature search was intentionally limited to the most recent 10-year period to ensure that the evidence reflects contemporary endodontic materials, irrigation protocols, antioxidant agents, and imaging methodologies relevant to current clinical and laboratory practice.

To build the search strategy, both MeSH terms and freely used keywords were combined to capture studies related to antioxidants and their various forms, sodium hypochlorite, sealer penetration, and dentinal tubules. Boolean operators (AND, OR) were used wherever needed Grey literature was also reviewed through Google Search to ensure that potentially relevant unpublished or work was not missed out.

### 2.2 Study eligibility

The eligibility criteria were conducted according to the preferred reporting items for systematic reviews and meta-analysis (PRISMA) guidelines and the Population, Intervention, Comparison and Outcome (PICO) design are as follows summarised in Table 2:

**Table 2:** PICO table.

Component	Description
Population (P)	Extracted human teeth with prepared root canals irrigated with sodium hypochlorite solution.
Intervention (I)	Application of antioxidant solution as a final irrigant solution.
Comparator (C)	Sodium hypochlorite-treated groups with no antioxidant treatment.
Outcome (O)	Depth of dentinal tubular penetration of sealer (µm) measured by confocal laser scanning microscopy (CLSM) or scanning electron microscopy (SEM)

Full-text manuscripts were reviewed and selected according to the following inclusion criteria:

1. *In vitro* studies evaluating the effect of antioxidant agents (such as ascorbic acid, sodium thiosulfate, gallic acid, tannic acid, or glutathione) on dentinal tubular penetration of endodontic sealers in sodium hypochlorite (NaOCl)–treated root canal dentin.
2. Studies including a control group that received NaOCl irrigation without subsequent antioxidant treatment.
3. Studies utilizing any resin-based, bioceramic, or calcium silicate–based sealer, and assessing penetration depth or interfacial adaptation by means of confocal laser scanning microscopy (CLSM), scanning electron microscopy (SEM), or other objective, reproducible microscopic evaluation methods supported by statistical analysis.
4. Studies published in peer-reviewed journals with full-text availability.

Parallelly, the following criteria were adopted as exclusion criteria for the qualitative synthesis:

1. Studies that were not laboratory-based, including clinical trials, animal experiments, or finite element analyses, were excluded.
2. Articles primarily evaluating bond strength, microleakage, or other physical/mechanical properties, without assessing sealer penetration or interfacial adaptation, were excluded.
3. Review articles, case reports, conference proceedings, editorials, and letters to the editor, were not considered eligible and were excluded.
4. Studies published in languages other than English were excluded.

### 2.3 Study selection

Studies were, screened initially by the title and abstract evaluation. Similarly, studies with deficient or inadequate information in the above mentioned sections were included for detailed review. Full text review of the articles were obtained and thoroughly examined independently by a single reviewer who decided which of the studies were included in this review.

### 2.4 Data collection process and data items

Relevant data were extracted from the four included studies using a standardized extraction form created in Microsoft Excel 2019. The extraction form captured the following items for each study: first author and year; country of origin; specimen type (human/bovine) and sample size; tooth preparation; irrigant used (type, concentration, exposure time and sequence); whether EDTA was used and when; antioxidant agent (type, concentration, exposure time and method of application); sealer/obturation material and obturation technique; outcomes assessed (dentinal tubular penetration, interfacial adaptation/gap analysis, push-out or other bond strength test); method of microscopic or mechanical evaluation (SEM, CLSM, UTM, push-out).

Before extraction, each study was checked against the review inclusion and exclusion criteria (*in vitro*; human-extracted teeth; NaOCl irrigation plus antioxidant applied as a final rinse; outcomes including tubular penetration, interfacial adaptation, or bond strength). The storage media and storage times for extracted teeth, were recorded.

Data extraction was carried out by a single reviewer, who collected all relevant information from the included studies in a structured manner. Any uncertainties that were encountered during extraction were resolved through checking of the full texts and verification against the predefined data items. Although contacting study authors for clarification was considered if needed, none of the included papers required additional communication to obtain the data used in this review.

All extracted numerical outcome data (means, SDs) were entered into the spreadsheet. When numerical data were presented graphically only, values were obtained from the figures using digital measurement tools where feasible and flagged in the extraction sheet as estimated.

### 2.5 Assessment of bias risk and quality of included studies

The methodological quality and risk of bias of all included studies were evaluated by one reviewer using a modified QUIN framework adapted for *in-vitro* dental research. The appraisal considered the reporting and/or execution of the following elements:

1. Clear statement of aims/objectives.
2. Description and justification of sample size (sample size calculation or rationale).
3. Description of specimen selection, inclusion/exclusion (sound, unrestored teeth) and storage method.
4. Randomization or allocation process for specimens into experimental groups.
5. Standardization of procedures (instrumentation, irrigation volumes/times, obturation technique).
6. Appropriateness of control groups.
7. Blinding of outcome assessor/operator of testing machine (where feasible).
8. Validated outcome assessment methods (SEM, CLSM, UTM) and description of measurement protocols.
9. Calibration of examiners or reporting of intra/examiner reliability (where applicable).
10. Appropriate statistical analysis and reporting (tests used, *p*-values).
11. Reporting of variability (SD or SEM) for quantitative outcomes.
12. Declaration of funding sources/conflicts of interest.

Each item was graded on a three-point scale: 2 when the criterion was fully met and clearly reported, 1 when it was only partly addressed, and 0 when it was not reported or inadequately covered. For studies that provided standard deviations for bond strength measurements, the coefficient of variation (CV = SD/mean) was calculated to judge variability. A low or moderate CV was considered acceptable and received a score of 2, while a high CV was scored as 0; borderline values were assigned 1.

The total score for each study (maximum possible score of 2 per item) was converted into a percentage to allow uniform interpretation across all included papers. Based on this percentage, studies were categorised as follows: 80% or above indicated low risk of bias; 60%–79% indicated moderate risk; and values below 60% were considered high risk.

The quality assessment was carried out by a single reviewer, who evaluated each study according to the predefined criteria.

## 3 Results

### 3.1 Study selection

**Figure 1:**
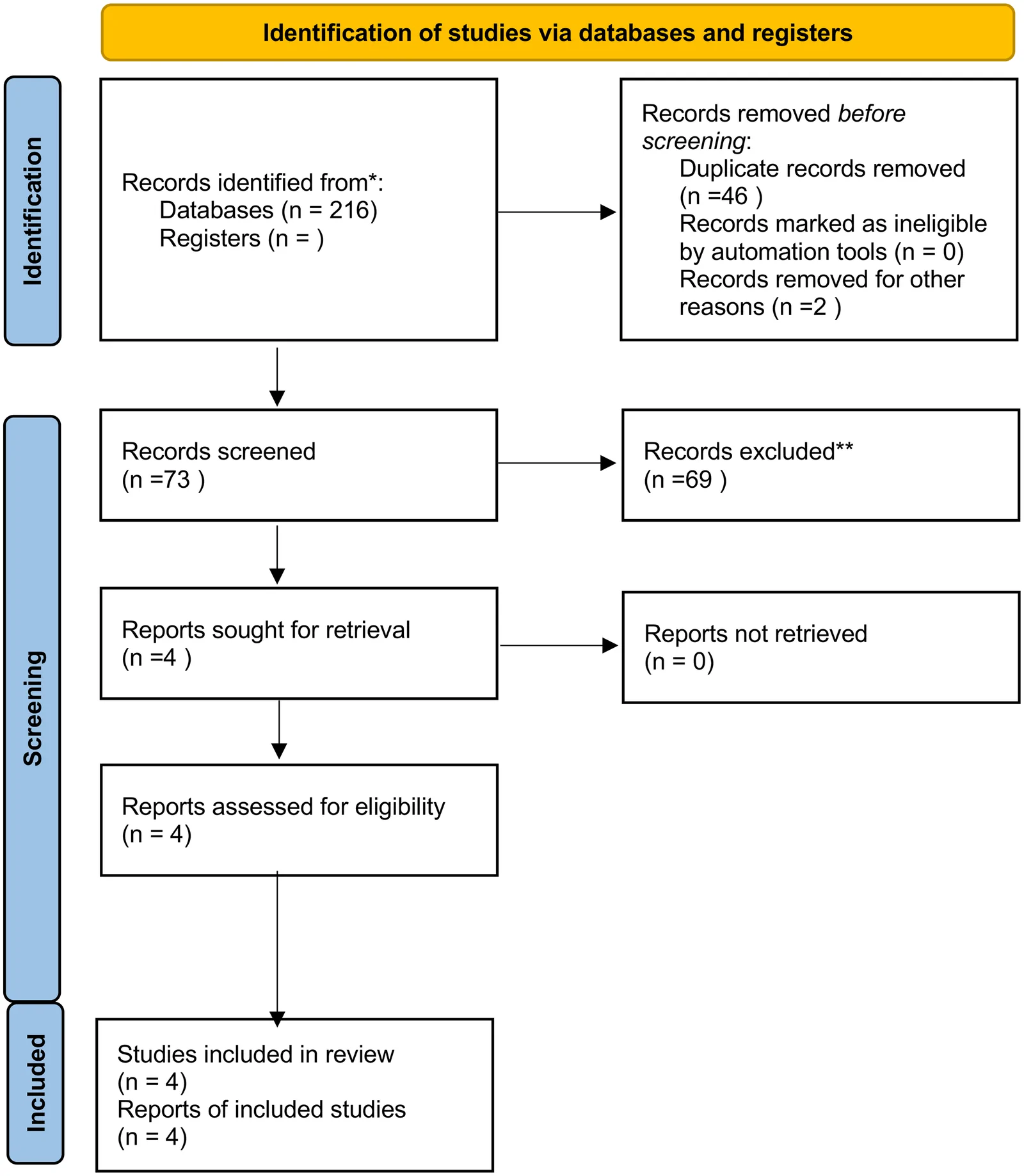
PRISMA flowchart.

Out of all the records retrieved, four *in-vitro* studies fulfilled the predefined eligibility criteria and were incorporated into the qualitative synthesis (7–10) according to PRISMA flowchart in Figure 1.

No additional studies were found through secondary or manual searching.

A narrative PRISMA flow summary is as follows: records identified (*n* = 4), records screened (*n* = 4), full-text articles reviewed (*n* = 4), and studies included in the final synthesis (*n* = 4). Reasons for excluding other retrieved items such as duplicate entries, irrelevant outcomes, animal experiments, and review papers were noted during the initial screening stage.

### 3.2 Study characteristics

Key study characteristics are summarized in Tables 3–6 and are summarized here.

- Christopher SR et al., 2016 (J Conserv Dent) (7). Country: India. Design: *in-vitro*. Sample: 50 human premolars decoronated to 14 mm. Irrigation: 5.25% NaOCl+ 17% EDTA (groups II–V). Antioxidants tested: 10% ascorbic acid, 10% tannic acid, 10% gallic acid (final rinse). Sealer: Resilon with RealSeal SE (methacrylate-based). Outcome: dentinal tubular penetration (SEM/CLSM), measured at cervical, middle, apical thirds. Main finding: gallic acid produced the greatest penetration (statistically significant vs. NaOCl control).
- Veenakumari R et al., 2019 (IJDSIR) (8). Country: India. Design: *in-vitro* SEM study. Sample: 40 human premolars decoronated to 16 mm. Irrigation: 5.25% NaOCl+ 17% EDTA (groups II–IV). Antioxidants: 10% tannic acid and 10% ellagic acid (final rinse). Sealer: AH Plus (epoxy resin). Outcome: SEM assessment of tubular penetration at coronal, middle, apical thirds. Main finding: ellagic acid produced superior penetration, especially in the coronal third.
- de Lima Dias-Junior LC et al., 2021 (Iran Endod J) (9). Country: Brazil. Design: *in-vitro*. Sample: 20 single-rooted human teeth prepared with ProTaper. Irrigation: 5.25% NaOCl ± 0.5% sodium thiosulfate (ST) as final rinse. Sealer: AH Plus labeled with rhodamine B for CLSM. Outcomes: interfacial adaptation (gaps/voids percentage) and dentinal sealer penetration (CLSM) at 2, 4, 6 mm sections. Main finding: NaOCl+ ST groups had fewer gaps/voids and greater penetration at coronal and middle thirds vs. NaOCl only.
- Charulatha RD et al., 2025 (Indian J Dent Sci) (10). Country: India. Design: *in-vitro*. Sample: 60 single-rooted premolars decoronated to 14 mm. Irrigation: 5.25% NaOCl ± glutathione (final rinse). Sealers: SafeEndo EpoxySeal (epoxy) and SafeEndo BioActive RCS (bioceramic). Outcomes: SEM for tubular penetration and push-out bond strength (UTM). Main finding: glutathione improved penetration for both sealers and significantly increased push-out bond strength (more pronounced for bioceramic sealer), shifting failure modes toward cohesive patterns.

**Table 3:** Data extraction Table 1.

No.	Study (author, year)	study objective	Sample (*N*, tooth type, preparation)	Irrigation protocol	Antioxidant used (concentration, duration, mode of application)	Sealer used (type, brand)	Evaluation method (microscopy, magnification, sections)	Outcome measures	Authors’ conclusions
1	Christopher SR et al., 2016 – Effect of three different antioxidants on the dentinal tubular penetration of Resilon and RealSeal SE on sodium hypochlorite-treated dentin	To compare the effect of ascorbic acid, gallic acid, and tannic acid on dentinal tubular penetration of RealSeal SE and Resilon after NaOCl irrigation.	50 extracted single-rooted human mandibular premolars; standardized root length; instrumented up to size F3; stored in saline.	Group I (negative control), irrigation was done with saline and 17% ethylenediaminetetraacetic acid (EDTA).For groups II (positive control), III, IV, and V, the irrigation protocol followed was 5 mL of 5.25% sodium hypochlorite between the change of each instrument and 5 mL of 17% EDTA for 1 min after biomechanical preparation. Final irrigation and flushing of the canals were done using distilled water.	10% Ascorbic acid(5 min), 10% Tannic acid(5 min), 10% Gallic acid (5 min)	RealSeal SE and Resilon cones	CLSM, 10 µm thick transverse sections at 2, 5, and 8 mm from apex. Fluorescent Rhodamine-B labeled sealer used.	Mean depth of sealer penetration (µm) and % area of penetration per section.	10% gallic acid was superior among the antioxidant irrigants that enabled the increased dentinal tubular penetration of Resilon and Real Seal SE.
2	Veenakumari R et al., (Year NR)–Comparative Evaluation of the Effect of Antioxidants on Dentinal Penetration of an Endodontic Sealer–An *in-vitro* SEM Study	The aim of this study is to evaluate the Effect 10% Tannic acid and 10% Ellagic acid on the dentinal tubular penetration of an endodontic sealer on sodium hypochlorite-treated root canal dentin.	Forty human premolars were decoronated to attain 16 mm root length and divided into four groups of 10 teeth each. Biomechanical preparation was done with rotary instruments.	Group I specimens were irrigated with saline and 17% ethylenediaminetetraacetic acid (EDTA). Specimens from groups II, III and IV were irrigated with 5.25% sodium hypochlorite and 17% EDTA.	Specimens from groups III and IV underwent additional irrigation with antioxidants– 10% Tannic acid and 10% Ellagic acid respectively.	AH Plus (epoxy resin-based)	SEM, longitudinal sections, ×1,500 magnification.	% of dentinal tubules showing sealer penetration; qualitative sealer adaptation score.	10% Ellagic acid was superior among the antioxidant irrigants compared with 10% Tannic acid that enabled the increased dentinal tubular penetration of endodontic sealer.
3	Charulatha RD et al., 2025–The Effect of Glutathione on Epoxy Resin-based and Bioceramic Sealers on Root Canal Dentin: An *in-vitro* Study	To evaluate the effect of glutathione on dentinal tubular penetration and push-out bond strength of SafeEndo epoxy resin sealer and SafeEndo bioceramic sealer in sodium hypochlorite (NaOCl)-irrigated root canal dentin.	60 single-rooted premolars were decoronated to 14 mm root length and divided into two groups (30 teeth each) based on the sealer type:	Group A (SafeEndo EpoxySeal) and Group B (SafeEndo BioActive RCS) which was subdivided as 1A and 1B as negative controls (saline), 2A and 2B as positive controls (NaOCl treated), and 3A and 3B as experimental groups (NaOCl followed by glutathione).	10% Glutathione, applied for 5 min as final irrigant.	SafeEndo epoxy resin sealer and SafeEndo bioceramic sealer	CLSM, 1 mm thick transverse sections at 2, 5, and 8 mm; Rhodamine B labeling.	Mean penetration depth (µm); interfacial gap measurements.	The bond strength of Epo-A shows the highest bond strength, and Bio-C, enhanced with glutathione. SEM indicates that Bio-C has superior cohesive integrity, minimizing microleakage risk.
4.	de Lima Dias-Junior LC et al., 2021–Effect of Sodium Thiosulfate on Interfacial Adaptation and Penetration of an Epoxy Resin-Based Root Canal Sealer	The study evaluated how the application of sodium thiosulfate as a final irrigant following sodium hypochlorite influences the quality of root canal obturation, specifically with respect to interfacial adaptation and sealer penetration of an epoxy resin–based root canal sealer.	20 single-rooted human teeth were prepared with the ProTaper system.	The specimens were divided into the following groups: 5.25% NaOCl irrigation (NaOCl group) and 5.25% NaOCl irrigation+0.5% sodium thiosulfate (NaOCl+ ST group).	5% Sodium thiosulfate for 3 min as a final rinse before drying and obturation.	AH-Plus sealer	Specimens were labeled with rhodamine B dye to allow analysis under a confocal laser scanning microscopy (CLSM). All samples were sectioned at 2, 4, and 6 mm from the apex and prepared for CLSM analysis.	The percentage of voids, gaps and dentinal sealer penetration segment of the canal were calculated at the apical, middle and coronal thirds.	The application of ST as an antioxidant agent after NaOCl irrigation showed better interfacial adaptation and penetration of epoxy resin-based root canal fillings.

**Table 4:** Data extraction Table 2.

Author (Year)	Country	Study design	Sample characteristics	Sealer evaluated	Microscopic evaluation
Christopher et al. (2016) (7)	India	*In-vitro*	50 extracted human mandibular premolars; decoronated to standardized length	RealSeal SE with Resilon cones	CLSM
Veenakumari et al. (2019) (8)	India	*In-vitro*	40 extracted human premolars; standardized root length	AH Plus	SEM
de Lima Dias-Junior et al. (2021) (9)	Brazil	*In-vitro*	20 extracted single-rooted human teeth	AH Plus	CLSM
Charulatha et al. (2025) (10)	India	*In-vitro*	60 extracted single-rooted human premolars; decoronated to 14 mm	SafeEndo EpoxySeal & SafeEndo BioActive RCS	CLSM, SEM

**Table 5:** Data extraction Table 3.

Author (Year)	NaOCl protocol	Antioxidant agent	Concentration	Application duration	Mode of application
Christopher et al. (2016) (7)	5.25% NaOCl+ 17% EDTA	Ascorbic acid, Tannic acid, Gallic acid	10%	5 min	Final irrigant rinse
Veenakumari et al. (2019) (8)	5.25% NaOCl+ 17% EDTA	Tannic acid, Ellagic acid	10%	Not specified	Final irrigant rinse
de Lima Dias-Junior et al. (2021) (9)	5.25% NaOCl	Sodium thiosulfate	0.5%	3 min	Final irrigant rinse
Charulatha et al. (2025) (10)	5.25% NaOCl	Glutathione	10%	5 min	Final irrigant rinse

**Table 6:** Data extraction Table 4.

Author (Year)	Primary outcome assessed	Key findings	Authors' conclusion
Christopher et al. (2016) (7)	Dentinal tubular penetration depth (µm)	All antioxidants improved penetration compared with NaOCl alone; gallic acid showed the highest penetration	Gallic acid was the most effective antioxidant for restoring dentinal tubular penetration
Veenakumari et al. (2019) (8)	Percentage of tubules penetrated	Ellagic acid showed superior penetration, particularly in the coronal third	Ellagic acid enhanced tubular penetration more effectively than tannic acid
de Lima Dias-Junior et al. (2021) (9)	Penetration segment and interfacial gaps	Sodium thiosulfate significantly reduced gaps and increased penetration at coronal and middle thirds	Sodium thiosulfate improved sealer penetration and interfacial adaptation
Charulatha et al. (2025) (10)	Penetration depth and push-out bond strength	Glutathione increased penetration and bond strength for both epoxy and bioceramic sealers	Glutathione effectively mitigated NaOCl-induced dentin alterations and enhanced sealer performance

### 3.3 Risk of bias (QUIN)

The modified QUIN assessment for the four included studies is summarized in Table 7. Using the 12-item binary scoring described above, the total scores and classifications were:

- Christopher et al., 2016 — total score 21/24 → 87.5% → Low risk of bias.
- Veenakumari et al., 2019 — total score 21/24 → 87.5% → Low risk of bias.
- de Lima Dias-Junior et al., 2021 — total score 21/24 → 87.5% → Low risk of bias.Charulatha et al., 2025 — total score 21/24 → 87.5% → Low risk of bias.
- All studies used validated outcome measures (SEM/CLSM/UTM) and reported variability measures (SD) for quantitative outcomes.

**Table 7:** Risk of bias.

QUIN Domain	Christopher 2016 (7)	Dias-Junior 2021 (9)	Charulatha 2025 (10)	Veenakumari 2019 (8)
1. Aim clearly stated	2	2	2	2
2. Sample size calculation	2	2	2	2
3. Sample selection & preparation described	2	2	2	2
4. Randomization of samples	2	2	2	2
5. Standardization of procedures	2	2	2	2
6. Control groups properly defined	2	2	2	2
7. Blinding of operator/examiner	0	0	0	0
8. Appropriate outcome assessment	2	2	2	2
9. Calibration of evaluator/instruments	1	1	1	1
10. Appropriate statistical analysis	2	2	2	2
11. Reporting of variability	2	2	2	2
12. Conflict of interest/funding declared	2	2	2	2
Total score (out of 24)	**21**	**21**	**21**	**21**

### 3.4 Results of individual studies

The following subsections summarize the main findings, organized by outcome domain.

#### 3.4.1 Dentinal tubular penetration

All four studies reported dentinal tubular penetration data (SEM or CLSM). The overall pattern was that NaOCl alone reduced tubular penetration relative to antioxidant-treated groups, and that antioxidant application restored or enhanced penetration.

Across studies, the magnitude of improvement varied by antioxidant and sealer chemistry; polyphenols (gallic/ellagic) produced large increases in penetration for resin sealers, sodium thiosulfate improved penetration and adaptation for epoxy sealers, and glutathione produced broad benefits for both epoxy and bioceramic systems.

#### 3.4.2 Interfacial adaptation and microscopic evaluation

Two studies reported quantitative or descriptive interfacial adaptation outcomes (9, 10). de Lima Dias-Junior et al. (9) documented a lower percentage of gaps/voids in NaOCl+ ST vs. NaOCl only groups at coronal and middle thirds (CLSM gap analysis). Charulatha et al. (10) reported smoother continuous interfaces and reduced interfacial gaps in glutathione groups by SEM. Visual inspection across the four studies consistently showed antioxidant-treated samples to possess open tubules, continuous sealer tags, and less collagen collapse compared with NaOCl-only samples.

#### 3.4.3 Bond strength (push-out/mechanical)

Only Charulatha et al. (10) performed push-out bond strength testing. Their reported results indicate that glutathione improved push-out bond strength for both tested sealers, with bioceramic sealer demonstrating stronger cohesive integrity following antioxidant treatment. Where comparative or baseline values were reported, NaOCl-only groups showed the lowest bond strengths.

#### 3.4.4 Failure mode

Failure mode was reported in Charulatha et al. (10): NaOCl-only groups exhibited predominantly adhesive failures, whereas antioxidant-treated groups shifted toward cohesive or mixed failures consistent with improved interfacial integrity and stronger sealer–dentin bonding.

## 4 Discussion

The dentinal tube penetration of endodontic sealers after NaOCl irrigation was assessed in all four included investigations, and significant variation was observed depending on the antioxidant and sealer type. NaOCl by itself consistently led to decreased tubular patency, surface collapse, and poor sealer penetration in every study. On the other hand, by correcting the oxidative changes to the dentin substrate caused by NaOCl, the subsequent application of antioxidants, such as tannic acid, ellagic acid, gallic acid, glutathione, and sodium thiosulfate, greatly increased dentinal penetration.

The impact of three antioxidants—10% ascorbic acid, 10% tannic acid, and 10% gallic acid—on the penetration of RealSeal SE sealer into dentinal tubules following exposure to 5.25% NaOCl was evaluated in the study by Christopher et al. (7). All antioxidants increased the dentinal tubular penetration as compared to NaOCl alone, according to SEM analysis. Gallic acid produced the highest penetration values, which supports its potent ability to scavenge polyphenolic free radicals.

Veenakumari et al. (8) assessed the dentinal tubular penetration of AH Plus sealer following the application of NaOCl and after final irrigation was done with either 10% tannic acid or 10% ellagic acid. According to SEM micrographs, ellagic acid outperformed tannic acid at all levels and produced the highest penetration in the coronal third, followed by the middle and apical thirds. Because of collagen collapse and reduced diameter of the tubules produced by NaOCl, NaOCl-only groups showed significantly lower penetration. Ellagic acid's strong polyphenolic antioxidant qualities and its capacity to maintain dentinal collagen architecture were cited by the authors as the reasons for its improved performance.

Charulatha et al. (10) evaluated the effects of 20% glutathione on the dentinal penetration of two sealers: SafeEndo BioActive RCS and SafeEndo EpoxySeal. According to CLSM analysis, glutathione significantly improved sealer penetration in both sealers when compared to NaOCl-only controls. Because of the bioceramic sealer subgroup's natural hydrophilicity and improved surface wetting following antioxidant treatment, the improvement was more noticeable. The authors highlighted that the thiol-containing tripeptide glutathione efficiently neutralizes reactive oxygen species (ROS) produced by NaOCl, restoring the redox state of the dentin and facilitating better sealer infiltration.

Using CLSM analysis, De Lima Dias-Junior et al. (9) examined 0.5% sodium thiosulfate following NaOCl irrigation and discovered a substantial increase in penetration depth at coronal and intermediate levels. When compared to NaOCl-only controls, the treated specimens showed fewer voids, continuous sealer interfaces, and open tubules. The mechanism underlying improved resin sealer penetration and restored dentin reactivity was identified as sodium thiosulfate's significant affinity for chlorinated oxidants.

Regardless of the type of sealer utilized, all experiments showed a comparable trend: antioxidants greatly enhanced dentinal tube penetration as compared to NaOCl alone.

All of the included investigations evaluated the quality of sealer adaptation to the canal wall using high-resolution microscopy (SEM or CLSM). NaOCl-treated dentin showed poor interfacial adaptation in all experiments, as evidenced by many voids, uneven resin tags, and surface imperfections.

According to Christopher et al. (7), CLSM showed that although NaOCl-only controls showed discontinuous sealer interfaces with dispersed gaps, antioxidant-treated groups showed uniform and continuous sealer penetration with well-formed resin tags.

According to Veenakumari et al. (8), SEM pictures of NaOCl-only specimens revealed destroyed tubules and collapsed collagen fibrils. On the other hand, the ellagic acid group produced the most continuous and densely packed sealer tags, whereas the tannic acid group showed greater sealer adaption and open tubules.

Improved interfacial adaptability in the glutathione-treated groups was further validated by Charulatha et al. (10). At the sealer–dentin interface, SEM showed better cohesive integrity, deeper sealer tag development, and fewer interfacial gaps, especially in the BioActive RCS group.

In a similar study, Dias-Junior et al. (9) found that as compared to the NaOCl-only group, the sodium thiosulfate treatment produces more consistent margins, continuous sealer flow, and fewer voids. Because of the increased surface energy and regenerated collagen, CLSM imaging showed smoother interfaces between the sealer.

Sodium hypochlorite in combination with EDTA remains the gold-standard irrigation protocol in endodontic practice due to its superior antimicrobial efficacy and smear layer removal capability. However, accumulating evidence from *in-vitro* studies indicates that these agents may adversely affect dentin by inducing collagen degradation, reducing microhardness, altering surface morphology, and creating an oxygen-rich substrate that can compromise the penetration and adaptation of resin-based root canal sealers. Such alterations may negatively influence the quality of the sealer–dentin interface and the long-term integrity of obturation.

The findings of the present review suggest that antioxidant agents have the potential to neutralize residual free radicals generated by sodium hypochlorite, thereby partially restoring the dentinal substrate and improving dentinal tubular penetration of root canal sealers. Although the available evidence is limited and derived exclusively from *in-vitro* studies, antioxidants may represent a promising adjunctive step aimed at mitigating the unfavorable effects of conventional irrigants on dentin.

In conclusion, microscopic examinations repeatedly showed that antioxidants successfully reverses the dentin changes caused by NaOCl, restores the surface wettability, resin infiltration, and overall the adaptability of both resin and bioceramic sealers.

## 5 Conclusion

Based on the limited *in vitro* evidence currently available, the findings of this review suggest that the use of antioxidant agents following sodium hypochlorite irrigation may help improve dentinal tubular penetration and interfacial adaptation of root canal sealers. The included studies indicate that antioxidants such as ascorbic acid and other naturally derived compounds are capable of reducing the adverse oxidising effects of sodium hypochlorite on dentin, resulting in better sealer interaction when compared with sodium hypochlorite treatment alone.

### 5.1 Limitations

1. This review included only four *in vitro* studies that fulfilled the eligibility criteria. Although these studies were systematically identified, the small volume of available evidence limits the strength of the conclusions that can be drawn from the present findings.
2. Noticeable differences existed among the included studies in terms of the antioxidants evaluated, the type of root canal sealers used, irrigation protocols, and outcome assessment methods. Such methodological variability made direct comparison difficult and did not allow for quantitative analysis.
3. All included investigations were conducted under *in vitro* conditions. While these models are useful for understanding dentinal interactions, they do not fully reflect the complexity of the clinical environment. As a result, any potential clinical relevance should be interpreted with caution.
4. The present review highlights the need for more well-designed studies with standardised methodologies, and future clinical investigations, to better clarify the role of antioxidants in influencing root canal sealer penetration and adaptation.

## Data Availability

The original contributions presented in the study are included in the article/Supplementary Material, further inquiries can be directed to the corresponding author.
